# The journal 'chiropractic & osteopathy' changes its title to 'chiropractic & manual therapies'. a new name, a new era

**DOI:** 10.1186/2045-709X-19-1

**Published:** 2011-01-10

**Authors:** Bruce F Walker, Simon D French, Melainie Cameron, Stephen M Perle, Charlotte Lebouef-Yde, Sidney M Rubinstein

**Affiliations:** 1 School of Chiropractic and Sports Science, Murdoch University, Western Australia, Australia; 2 Primary Care Research Unit, the University of Melbourne, Victoria, Australia; 3 School of Exercise Science, Australian Catholic University, Queensland, Australia; 4 College of Chiropractic, University of Bridgeport, Bridgeport, CT, USA; 5 Spinecenter of Southern Denmark, Hospital Lillebælt, Østre Hougvej 55, DK-5500 Middelfart, Denmark; 6 EMGO-Institute for Health and Care Research, VU University Medical Center, Amsterdam, The Netherlands

## Abstract

*Chiropractic & Osteopathy *changes its title to *Chiropractic & Manual Therapies *in January 2011. This change reflects the expanding base of submissions from clinical scientists interested in the discipline of manual therapy. It is also in accord with the findings of a review of the journal content and a joint venture between the original parent organisation the Chiropractic and Osteopathic College of Australasia and a new partner the European Academy of Chiropractic, which is a subsidiary body of the European Chiropractors' Union. The title change should encourage submissions from all professionals interested in manual therapy including chiropractors, osteopaths, physiotherapists, medical doctors and scientists interested in this field.

## 

This first paper in 2011 marks some significant changes for the journal. The first and most noticeable change is the title from *Chiropractic & Osteopathy *(C&O) to *Chiropractic & Manual Therapies (CMT)*. This change reflects the expanding base of submissions from clinical scientists interested in the discipline of manual therapy. It is also in accord with the findings and changes suggested by Coulter and Khorsan in their invited review of our journal. They noted that the articles in *C&O *are overwhelmingly from chiropractors and proposed several recommendations including a change in the journal title [1]. This title change does not exclude osteopathic submissions. To the contrary, the title change should encourage submissions from all professionals interested in manual therapy.

The decision to change the title was also influenced by successful negotiations with the European Academy of Chiropractic (EAC) to enter into a joint venture agreement on the journal. The EAC also encouraged a name change. This agreement with the EAC and its parent body the European Chiropractors' Union (ECU) was signed in June 2010 making the journal *Chiropractic & Osteopathy (C&O) *the official journal of the Chiropractic and Osteopathic College of Australasia (COCA) and the EAC.

COCA is a non-profit, member-based organisation that provides continuing education to its members predominantly in Australia (COCA website [2]). COCA aims to assist all members of the chiropractic, osteopathic and related health professions to engage with best practice healthcare methods and to develop the skills required to practice competently. COCA encourages a scientific and ethical approach to patient management, fosters related research, and seeks to participate in activities related to public health with an emphasis on promoting the integration of chiropractors and osteopaths into the broader community healthcare community. Given the broad goals of COCA the name change of its journal seems to fulfil those aims.

The ECU is a Union of nineteen European chiropractic associations from Belgium, Cyprus, Finland, France, Germany, Great Britain, Greece, Iceland, Ireland, Italy, Liechtenstein, Luxembourg, The Netherlands, Norway, Poland, Portugal, Spain, Sweden, and Switzerland (ECU website [3]). The ECU was established to promote the development of chiropractic in Europe as well as to pursue the interests of chiropractic as a science and a profession by research, teaching, publications and legal activities.

The EAC's purpose is to act as the academic arm of its parent organisation the ECU. It is responsible for the academic promotion of the chiropractic profession in Europe and liaises with other academic institutions (EAC website [4]).

There were several reasons to pursue a joint venture; first were economies of scale regarding funding for the journal with EAC now contributing half the expenses incurred. This has allowed COCA and EAC to cover the cost of article-processing charges for all manuscripts submitted before April 2011. This will enable *Chiropractic & Manual Therapies *to remain an international open access journal without charge to authors during this time. Further extension of this charging policy will be discussed in early 2011. This is unusual as the vast majority of online journals charge authors to publish. The second reason to pursue a joint venture was that it provides a broader international supporter base for the journal, providing wider readership and an anticipated increase in the number of high quality manuscripts submitted. Since 2005 the journal has received over 320 submissions and accepted 130 of these for publication, rejecting or suggesting withdrawal of approximately 60% mainly due to poor methodological and scientific quality found at peer review.

The joint venture and re-naming of the journal enhances it as a major international publication by means of greater global acceptance and marks the start of the next phase of growth of the journal including application for an impact factor and MEDLINE listing.

There have also been key changes to the journal's editorial team with two new Associate Editors from Europe joining the team. Professor Charlotte Leboeuf-Yde (Denmark) and Dr Sidney Rubinstein (The Netherlands) add to the depth and breadth of the existing Associate Editorial team which includes Dr Simon French and Associate Professor Melainie Cameron (Australia), and Professor Stephen Perle (USA). The position of Editor-in-Chief remains unchanged with Dr Bruce Walker in Australia. The Editorial Board has 42 members and is extensive in its skills, experience and diversity [5].

Given these historic changes, we predict an increase of high quality submissions over the coming years with continued growth and international positioning of our newly named journal "*Chiropractic & Manual Therapies*". We look forward to your comments as readers and your submissions as authors.
